# Architecture of an Antagonistic Tree/Fungus Network: The Asymmetric Influence of Past Evolutionary History

**DOI:** 10.1371/journal.pone.0001740

**Published:** 2008-03-05

**Authors:** Corinne Vacher, Dominique Piou, Marie-Laure Desprez-Loustau

**Affiliations:** 1 Institut Scientifique de Recherche Agronomique (INRA), UMR1202 Biodiversité Gènes et Communautés, Villenave d'Ornon, France; 2 Département de la Santé des Forêts, Ministère de l'Agriculture et de la Pêche, Cenon, France; Centre National de la Recherche Scientifique, France

## Abstract

**Background:**

Compartmentalization and nestedness are common patterns in ecological networks. The aim of this study was to elucidate some of the processes shaping these patterns in a well resolved network of host/pathogen interactions.

**Methology/Principal Findings:**

Based on a long-term (1972–2005) survey of forest health at the regional scale (all French forests; 15 million ha), we uncovered an almost fully connected network of 51 tree taxa and 157 parasitic fungal species. Our analyses revealed that the compartmentalization of the network maps out the ancient evolutionary history of seed plants, but not the ancient evolutionary history of fungal species. The very early divergence of the major fungal phyla may account for this asymmetric influence of past evolutionary history. Unlike compartmentalization, nestedness did not reflect any consistent phylogenetic signal. Instead, it seemed to reflect the ecological features of the current species, such as the relative abundance of tree species and the life-history strategies of fungal pathogens. We discussed how the evolution of host range in fungal species may account for the observed nested patterns.

**Conclusion/Significance:**

Overall, our analyses emphasized how the current complexity of ecological networks results from the diversification of the species and their interactions over evolutionary times. They confirmed that the current architecture of ecological networks is not only dependant on recent ecological processes.

## Introduction

A network is a set of items, called *vertices* (or *nodes*), connected by *edges* [1]. Networks have been used to portray the complexity of systems in various fields of research. In ecology, networks are a valuable tool for representing the diversity of species (*vertices*) and their interactions (*edges*). They were originally used for studies of predator/prey interactions (food webs), and have more recently been used for studies of long-lasting, ‘intimate’ interactions [2] between two sets of species (e.g. plant species and their pollinators or host species and their parasites). Unlike food web networks, plant/pollinator and host/parasite networks have two types of vertices (each type representing one set of species) with edges connecting vertices of unlike type only. These networks are therefore described as *bipartite* networks [1] and are commonly depicted with a two-layer graph or a binary matrix [3].

Two patterns have repeatedly been found in bipartite networks of species interactions: nestedness and, to a lesser extent, compartmentalization. A nested network displays both asymmetric specialization—i.e. species with few interactions (‘specialist’ species) preferentially interact with species with many interactions (‘generalist’ species)— and a dense core of interactions created by symmetric interactions between generalist species. Significant nested structures have been found in many bipartite mutualistic networks, including plant/pollinator networks [4], plant/seed disperser networks [4], ant/plant networks [5], fish cleaning symbiosis [6] and anemone fish/anemone networks [7]. They have also been found in some bipartite antagonistic networks, such as plant/phytophagous insect networks [3]. Recent findings indicate that this pervasive pattern is a relatively robust measure of network structure, less prone to variations of sampling effort in space and time than number of species and links within the network [8]. The ecological and evolutionary processes having shaped this pattern are currently a matter of debate [9]–[11].

Compartmentalization is characterized by recognizable subsets of interacting species, with species more likely to be linked within than across subsets. No consensus on its prevalence in ecological networks has yet been reached [12]. It has been found in a few bipartite networks, including plant/phytophageous insects networks [13], plant/ant networks [14], [15] and plant/pollinator networks [12], [16], and seems more frequent in networks of large size [12]. Several processes have been identified as potentially playing a role in the emergence of compartmentalization. On the ecological timescale, compartmentalization may arise through spatial [17] or temporal [16] segregation of the species. Species occurring in the same place, at the same time are more likely to fall into the same compartment, because they have a higher probability of interacting with each other than with species occurring elsewhere or at another time. However, compartmentalization may also reflect more ancient events, such as phylogenetic splits [3], [14], [18]. Typically, a pollinator's diet is constrained by its phylogenetic origin. For example, dipteran pollinators do not have long enough tongues to reach the nectar in tubular flowers, whereas lepidopteran pollinators do. Thus, the insects of the Diptera and Lepidoptera families tend to fall into different compartments, corresponding to open and tubular flowers, respectively [16].

In this study, we described a well resolved network of host/pathogen interactions in the forest ecosystem. We addressed the following questions: Are there significant compartments and nested structures in this antagonistic web of species? Which ecological and evolutionary processes have shaped these patterns? Based on a long-term (1972–2005) survey of forest health at the regional scale (all French forests; 15 million ha), we uncovered an almost fully connected network of 51 tree taxa and 157 parasitic fungal species. We analysed the influence of several species traits (phylogeny, abundance, distributional range, life-history strategy) on their position in the network architecture. Our analyses showed that there are still signs of the ancient evolutionary history of the species in the current network architecture.

## Materials and Methods

### Tree-fungus interaction records

For this study, we used a compilation of 11087 records of forest tree diseases caused by parasitic fungi. All the observations originated from the database of the *Département Santé des Forêts* (DSF) for the 1972–2005 period. DSF is the French governmental organization in charge of forest health monitoring. It consists of a network of skilled foresters evenly covering the state-owned and private forests of the country. The primary aim of the DSF network is to prevent disease spread, pest outbreaks and other types of damage by alerting the authorities as soon as a threat to forest health is identified. The foresters report all types of damage noticed during their daily work in the forest, when they consider that they may reduce the survival or the economical value of trees. Their reports contain the full description of the observed symptoms and, when applicable, the *in situ* identification of the biotic agent which is responsible for them. When the *in situ* morphological identification is tricky, samples are sent to the National Plant Protection Laboratory (LNPV) which specializes in the molecular identification of plant pests and pathogens. When the damage of a forest plant has several causes (for instance, an insect attack followed by the spread of a fungal parasite), all of them are recorded and ranked in their order of importance. Hence, pests and pathogens recorded in the database are not only primary pests and pathogens but also secondary or weakness pests and pathogens. Over 67,000 cases of insect attack, fungal disease, abiotic stresses or decline were reported between 1972 and 2005.

For this study, we only used observations in which fungi had been identified to the species level. We updated fungal species names and corrected species synonyms with the *Index Fungorum* database (www.indexfungorum.org). We obtained a database of 157 fungal species (see Table S1 in Supplementary Material) and 51 host taxa (see Table S2), forming 547 interactions. These data will be uploaded to the Interaction Web Database (www.nceas.ucsb.edu/interactionweb/).

### Fungal species traits

#### Phylogeny

All the fungal species belonged to Dikarya, which consists of two monophyletic phyla: Ascomycota and Basidiomycota. Ascomycota is the largest phylum and is divided into three monophyletic subphyla: Taphrinomycotina, Saccharomycotina and Pezizomycotina. Basidiomycota is also divided into three subphyla: Pucciniomycotina, Ustilagomycotina and Agaricomycotina [19]. Based on the *Index Fungorum* database (www.indexfungorum.org) and the NCBI taxonomy browser [20], we classified the fungal species into phyla and subphyla. Only three species could not be assigned to a subphylum (Table S1).

#### Life-history strategy

We also classified the fungal species into 10 nutritional types (referred to as ‘life-history strategies’, as suggested by Garcia-Guzman and Morales [21] based on the parasitic lifestyle (biotroph versus necrotroph) and on the plant organs and tissues attacked: (1) strict foliar necrotroph parasites, (2) canker agents, (3) stem decay fungi, (4) obligate biotroph parasites, (5) root decay fungi, (6) other foliar and twig necrotroph parasites, (7) stem blue stain agents, (8) parasites of fine roots, (9) wilting agents, (10) other root fungi. We included only the five first strategies, which accounted for 87% of the fungal species, in statistical analyses.

### Tree species traits

#### Phylogeny

Of the 51 tree taxa, 41 corresponded to true species, 4 corresponded to groups of cultivars belonging to the same genetic continuum and 6 corresponded to groups of several species belonging to the same genus (Table S2). The tree species were equally distributed between two phyla of Division Spermatophyta: the Magnoliophyta and the Coniferophyta. We used the Angiosperm Phylogeny Website [22] to classify the tree taxa further, into seven subphyla (Proteales, Malpighiales, Fabales, Rosales, Fagales, Malvales, Sapindales, Lamiales and Pinales). Moreover, phylogenetic distances between the species belonging to the Magnoliphyta (angiosperms) were estimated by using the Phylomatic software [23]. This tool allowed us to create an hypothesis for the phylogenetic relationships among the tree species based on the dated angiosperm supertree of Davies and collaborators [24]. The resultant tree was ultrametric, with branch length reflecting estimated time between branching events. Pairwise phylogenetic distances were then extracted by using the *cophenetic.phylo* function of the R ape package [25].

#### Abundance and sampling intensity

An estimate of area covered by each tree taxon (hereafter called abundance) was available (*Inventaire Forestier National*, 2000 census report). An estimate of the total number of times each tree taxon had been encountered and examined by foresters during their daily work was also available from the DSF database. This number, further referred to as sampling intensity, was expected to be highly correlated with abundance. We assumed that over the long period of the survey (1972–2005) and over the large geographical scale considered here (the entire French territory), the probability for a tree taxa of being damaged was on average equal for all tree taxa. We therefore defined the sampling intensity of a tree taxon as the total number of DSF records for all types of damage (i.e. damage caused by insects, mammals, human activities or abiotic stresses). Damage caused by parasitic fungi were excluded from the calculation in order to obtain an estimate of sampling intensity independent from the data analysed in this study (i.e. tree/fungus interaction records).

#### Distributional range

The *Inventaire Forestier National*, the French agency responsible for monitoring forest productivity and composition, has divided the country into 309 geographical units, each of which is homogeneous in terms of its climate, soil and relief. Presence/absence data for all the tree taxa except one (*Pinus radiata*) were available for these 309 geographical units (*Inventaire Forestier National*, 2000 census report) and were used to estimate the distributional overlap between tree taxa.

### Detection of compartmentalization and statistical validation

#### Detection

We first identified the connected components of the interaction network and characterized them in terms of size, using the *clusters* function of the R igraph package [26]. The connected components of the network represent, in grossest terms, the pieces of the network: two vertices are in the same component if and only if there is some path between them. We defined the size of a component as the number of its vertices. We then applied the clustering algorithm proposed by Girvan and Newman [27] to the largest component, to highlight its structure. As described by Girvan and Newman [27], we first calculated betweenness for all edges of the largest component, using the *edge.betweenness* function of the R igraph package [26]. Betweenness, which was originally defined for graph vertices [28], [29] and was then extended to graph edges [27], is approximately equal to the number of shortest paths going through a vertex or an edge. As described by Girvan and Newman [27], if a network contains clusters that are loosely connected by a small number of edges, then edges connecting clusters have a high edge betweenness, because all shortest paths between different clusters must go along these edges. Edges with the highest betweenness were therefore removed from the graph, and we recalculated betweenness for all the remaining edges [27]. This sequence was repeated until the clusters were separated. Each cluster was then split in turn, starting with the largest. The algorithm was repeated until no edges remained. The nested hierarchy of clusters was converted into a tree format, using the *as.phylo.formula* function of the R ape package [25]. The hierarchical tree was represented with TreeView [30]. The order of tree leaves on the hierarchical tree was used to reorder the rows and columns of the interaction matrix.

#### Statistical validation

The clusters were validated statistically by testing whether element similarity (i.e. similarity between fungal species as a function of host and similarity between host taxa as a function of their pathogens) was significantly higher within than between clusters. We used the multiresponse permutation procedure (MRPP), a non parametric test of differences between predefined groups [31]. The MRPP statistics *δ* is the weighted mean of within-group means of pair-wise dissimilarity between group elements. As described by Prado and Lewinshon [13], dissimilarity was calculated as a Jaccard distance and group size was taken as the group weight. We used the *mrpp* function of the R vegan package [32] to calculate the expected statistics E(*δ*) if groups were assembled at random. The within-group chance-corrected agreement (A), defined as 1- *δ*/E(*δ*), has a maximum of 1 when there is no dissimilarity between the elements of any group. The P-value is the probability of obtaining, by chance, a value of A equal to or larger than the observed value.

### Detection of nestedness and statistical validation

#### Detection

Nestedness was defined as N = (100-T)/100, where T is the matrix temperature—a measure of matrix disorder with values ranging from 0° (perfectly nested) to 100° (perfectly non nested) [4]. Values of N close to 1 therefore indicate a high degree of nestedness. The adjacency matrix was maximally packed to calculate T (see the article by Atmar and Patterson [33] for further details). An isocline of perfect nestedness was then calculated and deviations from this isocline (i.e. unexpected presences and absences of interactions deviating from a perfectly nested pattern) were scored. Matrix temperature T is the average degree of deviation from this isocline. All nestedness analyses were performed with an improved version of Nestedness Calculator software [33] called ANINHADO v.2.03. [34].

#### Statistical validation

The significance of nestedness was assessed using two null models. Null model I is the null model implemented in Nestedness Calculator software [33]. It assumes that each cell of the interaction matrix has the same probability of being occupied. This probability is estimated as the number of “1s” in the matrix divided by the number of cells. This model generates networks in which differences in the number of interactions between species are small. Hence, deviations from this null model may be due to both differences in the number of interactions between species and an asymmetric distribution of interactions between species. Null model II was developed by Bascompte and collaborators [4] to cope with this problem. It assumes that the probability of each cell being occupied is the average of the probabilities of occupancy of its row and column. Hence, deviations from this null model result solely from an asymmetric distribution of interactions between species. For each type of null model, a population of 1000 random networks was generated using ANINHADO software [34]. The P-value is the probability of a random replicate being at least as nested as the observed matrix.

### Influence of species traits on their position in the compartmentalized network

#### Phylogeny of tree and fungal species

We first performed Monte Carlo tests with 10000 replicates, using the *chisq.test* function of the R stats package [35], to assess whether the distribution of tree species and fungal species in the different network compartments was random with respect to their phyla and subphyla. In the case of tree species, we also performed the multiresponse permutation procedure (MRPP) to test whether the phylogenetic distance between species was significantly lower within than between compartments, by using the *mrpp* function of the R vegan package [32] and by taking group size as the group weight.

#### Distributional range of tree species

Then we performed the multiresponse permutation procedure (MRPP), by using the *mrpp* function of the R vegan package [32], to test whether the dissimilarity between tree species in their distributional ranges was significantly lower within than between compartments. The dissimilarity between two species in their distributional range was defined as the Jaccard distance between their presence/absence vectors [36]. A distance of zero indicates that the two species have identical ranges, whereas a distance of one indicates that ranges do not overlap at all. As previously, group size was taken as group weight in the permutation procedure.

#### Life-history strategies of fungal species

Finally we investigated whether tree taxa from different compartments were linked by fungal species having a particular life-history strategy in common, by calculating the number of tree groups linked together by each fungal species and comparing this number for different strategies, using Kruskal-Wallis rank sum tests, implemented with the *kruskal.test* function of the R stats package [35].

### Influence of species traits on their position in the nested network

Species position in the nested network was defined as their rank in the interaction matrix when reorganized for nestedness. We first assessed the relationships between the total number of interactions per species and their rank with Kendall's rank correlation tests, performed with the *cor.test* function of the R stats package [35], in order to verify that low-ranked species were those belonging to the dense core of interactions of the nested network. Then we characterized the core tree species by their phylum, abundance and sampling intensity and the core fungal species by their phylum and life-history strategy.

#### Phylogeny of tree and fungal species

We compared mean species rank as a function of phylum, using Wilcoxon rank sum tests, carried out with the *wilcox.test* function of the R stats package [35].

#### Abundance and sampling intensity of tree species

The relationships between the abundance of a given tree taxon and rank in the nested structures were assessed with Kendall's rank correlation tests, performed with the *cor.test* function of the R stats package [35]. The same test was performed to assess the relationships between the sampling intensity of a given tree taxon and rank in the nested structures.

#### Life-history strategies of fungal species

We performed Kruskal-Wallis rank sum tests, implemented with the *kruskal.test* function of the R stats package [35], to compare the rank of fungal species in the nested structures for the different life-history strategies.

## Results

**Figure 1:**
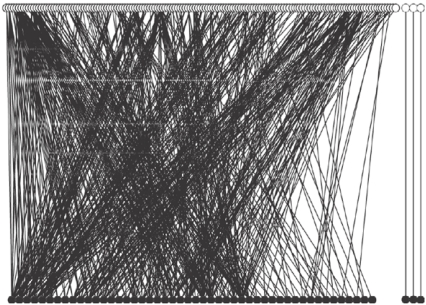
Architecture of the tree-fungus network. Black circles correspond to tree species whereas white circles correspond to fungal species. The network was drawn with PAJEK software (http://vlado.fmf.uni-lj.si/pub/networks/pajek/).

Most of the parasitic fungal species were highly specialized (57% of the species had only one or two host taxa), but the tree/fungus interaction network was almost fully connected. Only three pair-wise host-parasite interactions were isolated from the largest connected component, which consisted of 48 host taxa, 154 fungal species and 544 interactions (Fig. 1).

### Isolated, pair-wise interactions

One of the three isolated interactions involved yew (*Taxus baccata*) and the parasite species *Phomopsis juniperivora*, consistent with previous reports showing that yew is affected by few serious fungal diseases [37], [38]. Another isolated interaction was that between elm (*Ulmus sp.*) and *Ophiostoma novo-ulmi* (grouped with *O. ulmi*), the causal agent of Dutch elm disease. The dramatic consequences of this disease in Europe [39] may account for foresters involved in forest health monitoring not focusing on the other diseases that occur on elm. The third isolated interaction involved birch (*Betula sp.*) and the parasite species *Taphrina betulina*. This isolation resulted from a recording bias. Birch may actually be linked to the main network component through interactions with *Armillaria* species [40].

### Compartmentalization

#### Detection and statistical validation

**Figure 2:**
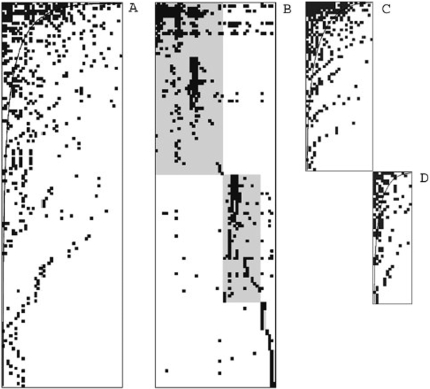
Structure of the largest connected component of the tree/parasitic fungus interaction network. (A) Matrix ordered for nestedness. (B) Matrix ordered for compartmentalization. Six compartments are aligned on the first diagonal of the matrix. These compartments, C1 to C6, run from the bottom right corner to the upper left corner. C1, C4, C5 and C6 are highlighted in gray. (C) Compartment C5 rearranged for nestedness. (D) Compartment C6 rearranged for nestedness.

Sequential splitting of the largest component of the network with the edge betweenness algorithm [27] led to the identification of six compartments (Fig. 2B), which were validated statistically (Table 1). The first split of the network produced two groups of unequal size. The smallest compartment (C1) contained two host taxa (*Populus tremulae* and cultivated poplars) and 15 fungal species, whereas the largest group consisted of 46 host taxa and 139 fungal species. The first split of this larger group also yielded two groups of unequal size, the smallest (C2) containing one host taxon (*Castanea sativa*) and six fungal species. Subsequent splits of the largest group produced compartment C3, consisting of one host taxon (*Tilia* spp) and two fungal species, and compartment C4, containing two host taxa (*Acer* spp) and 11 fungal species. The fifth split of the network yielded groups of almost equal size: C5 and C6. C5 was composed of 15 host taxa and 51 fungal species, whereas C6 was composed of 27 host taxa and 69 fungal species. The interaction dissimilarity between fungal species was higher within these two large compartments than within the smaller compartments C1 to C4 (Table 1). We therefore assessed the significance of the fifth network split for fungal species, by excluding the species of compartments C1 to C4. The result remained significant, despite the decrease in the index of within-group agreement (MRPP; A = 0.069; p-value<0.001).

**Table 1:** Multiresponse permutation procedure (MRPP) analysis of group dissimilarities, showing mean parasite dissimilarity between hosts and mean host dissimilarity between parasites in each compartment of the largest connected component of the tree-parasitic fungi network.

	Intragroup mean Jaccard distance					
	Between hosts as a function of their parasites			Between parasites as a function of their hosts		
Compartment	Group Size		Distance	Group Size		Distance
C1	2		0.92	15		0.39
C2	1		-	6		0.22
C3	1		-	2		0.00
C4	2		0.78	11		0.25
C5	15		0.76	51		0.85
C6	27		0.95	69		0.88
Intragroup agreement (A)		0.047			0.213	
*p*-value		<0.001			<0.001	

The chance-corrected agreement index (A) reflects within-group homogeneity and has a maximum value of 1 when there is no dissimilarity between the elements of any group. The *p*-value is the probability of obtaining, by chance, a value of A at least as high as the observed value.

#### Relationships with the phylogeny of tree species

**Figure 3:**
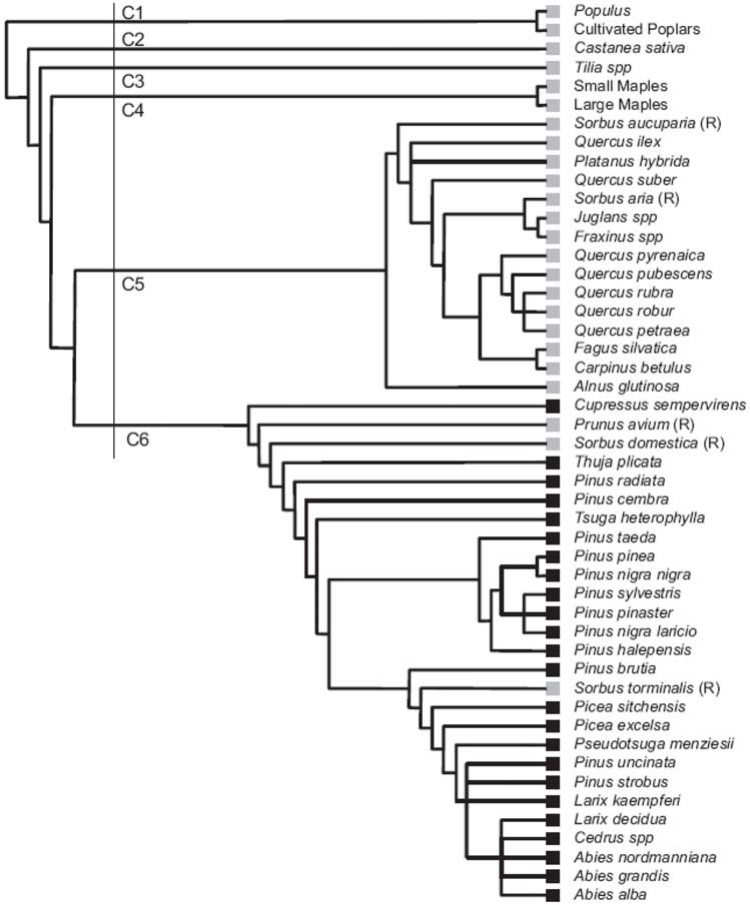
Hierarchical tree showing the sequential splits of the largest connected component of the tree/parasitic fungus network. Due to space constraints, only tree taxa are represented. Branches corresponding to the six network compartments (C1 to C6) are indicated. Tree leaf symbols correspond to tree phyla (gray squares: Magnoliophyta; black squares: Conipherophyta). R denotes tree species of the Rosaceae family.

All the host taxa of compartments C1 to C5 belonged to the Magnoliophyta, whereas all but three of the taxa in C6 belonged to the Conipherophyta (Fig. 3). Interestingly, the three host taxa from the Magnoliophyta that fell within the C6 group (*Prunus avium*, *Sorbus domestica* and *Sorbus torminalis*) were all from the Rosaceae family—a well supported branch of the Rosales subphylum [22]. The host taxa of the C1 compartment were the only two host taxa from the Malpighiales subphylum; the host taxon of the C3 compartment was the only host taxon from the Malvales subphylum, and the host taxa of the C4 compartment were the only host taxa of the Sapindales subphylum. Thus, tree taxa were not randomly distributed within the compartments in terms of their phylogenetic origin (Monte Carlo test; phylum: p-value<0.001; subphylum: p-value<0.001). Phylogenetic distance analyses confirmed that the taxa belonging to the Magnoliophyta were not distributed at random within the compartments C1 to C6 (MRPP; A = 0.324; p-value<0.001).

#### Relationships with the distributional range of tree species

The distribution of tree taxa between compartments C1 to C6 in terms of their distributional range was also significantly different from random (MRPP; A = 0.042; p-value = 0.003). The high distributional overlap of the two taxa belonging to the small compartment C4 (*Acer* species) and, to a lesser extent, that of the two taxa belonging to the small compartment C1 (*Populus* species) may account for this result (mean Jaccard distance; 0.54, 0.15, 0.75, 0.87 for compartments C1, C4, C5 and C6, respectively). We therefore performed again the analysis, but only for the large compartments C5 and C6. The distribution of tree taxa in terms of their distributional range was then marginally significantly different from random (MRPP; A = 0.007; p-value = 0.087). Finally we excluded the three species belonging to the Rosaceae family from the C6 compartment to investigate whether the emergence of the large compartments of the network may have been driven by the spatial segregation between Conipherophyta and Magnoliophyta. The index of within-group agreement increased and the statistics remained marginally significant (MRPP; A = 0.010; p-value = 0.056).

#### Relationships with the phylogeny of fungal species

The distribution of fungal species between compartments was random with respect to subphylum (Monte Carlo test; p-value = 0.219), but there was a slight deviation from randomness for phylum (Monte Carlo test; p-value = 0.043), with the small compartments—C1 to C4—having a higher proportion of Ascomycota than would be expected if the distribution were random (90.2% *versus* 69.5%).

#### Relationships with the life-history strategy of fungal species

**Figure 4:**
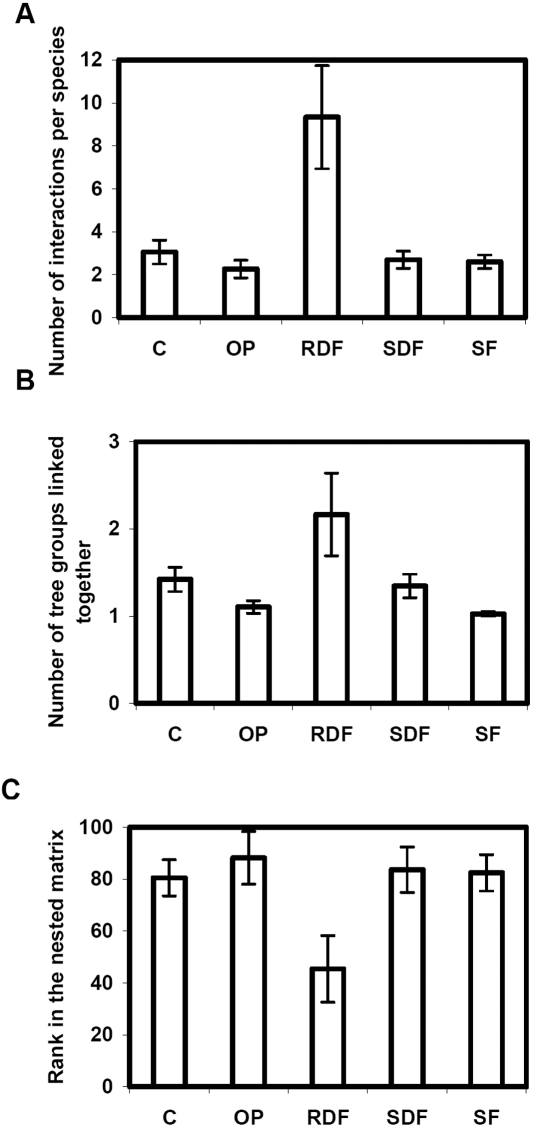
Comparison of the major pathogenic types (C: canker agents, OP: obligate biotroph parasites, RDF: root decay fungi, SDF: stem decay fungi, SF: strict foliar necrotroph parasites). Bars indicate standard errors of the means. (A) For the number of interactions per species. (B) For the number of tree groups linked together. Two tree taxa belong to the same group if they belong to the same network compartment. (C) For the rank in the network's largest connected component after rearrangement for nestedness.

Life-history strategy had a significant effect on the total number of interactions per fungal species (Kruskal-Wallis rank sum tests; χ^2^ = 11.5, df = 4, p-value = 0.021) and the number of tree groups linked together by fungal species (Kruskal-Wallis rank sum tests; χ^2^ = 21.5, df = 4, p-value<0.001). Root decay fungi had the largest host range: they had the highest mean number of host taxa (Fig. 4A) and linked together the largest number of network compartments (Fig. 4B).

### Nestedness

#### Detection and statistical validation

The largest component of the network showed significant nestedness (N = 0.900; p-value<0.001 both for null models I and II) (Fig. 2A). Compartment C5 was significantly nested (N = 0.775; p-value<0.001 for null model I; p-value = 0.002 for null model II) (Fig. 2C), as was compartment C6 (N = 0.870; p-value<0.001 both for null models I and II) (Fig. 2D). As expected, the number of interactions per species and their rank in the nested structures were significantly and negatively correlated, both for tree species (Kendall's rank correlation test; τ = −0.90; p-value<0.001 for the network largest component; τ = −0.91; p-value<0.001 for compartment C5; τ = −0.90; p-value<0.001 for compartment C6) and fungal species (Kendall's rank correlation test; τ = −0.86; p-value<0.001 for the network largest component; τ = −0.79; p-value<0.001 for compartment C5; τ = −0.88; p-value<0.001 for compartment C6). Hence, low-ranked species had more interactions than high-ranked species and belonged to the core of the nested structures.

#### Relationships with the phylogeny of tree and fungal species

The ranks of the tree taxa belonging to Conipherophyta and Magnoliophyta phyla were similar in the largest network component (Wilcoxon rank sum test; W = 291; p-value = 0.959), as were the ranks of the fungal species belonging to the Ascomycota and Basidiomycota phyla (Wilcoxon rank sum test; W = 2914, p-value = 0.117). The ranks of fungal phyla differed slightly between compartments C5 (Wilcoxon rank sum tests; W = 229, p-value = 0.080) and C6 (Wilcoxon rank sum tests; W = 633, p-value = 0.059). In compartment C5, fungal species belonging to the Ascomycota had a tendency to have lower ranks whereas the opposite trend was found in compartment C6.

#### Relationships with the abundance and sampling intensity of tree species

**Figure 5:**
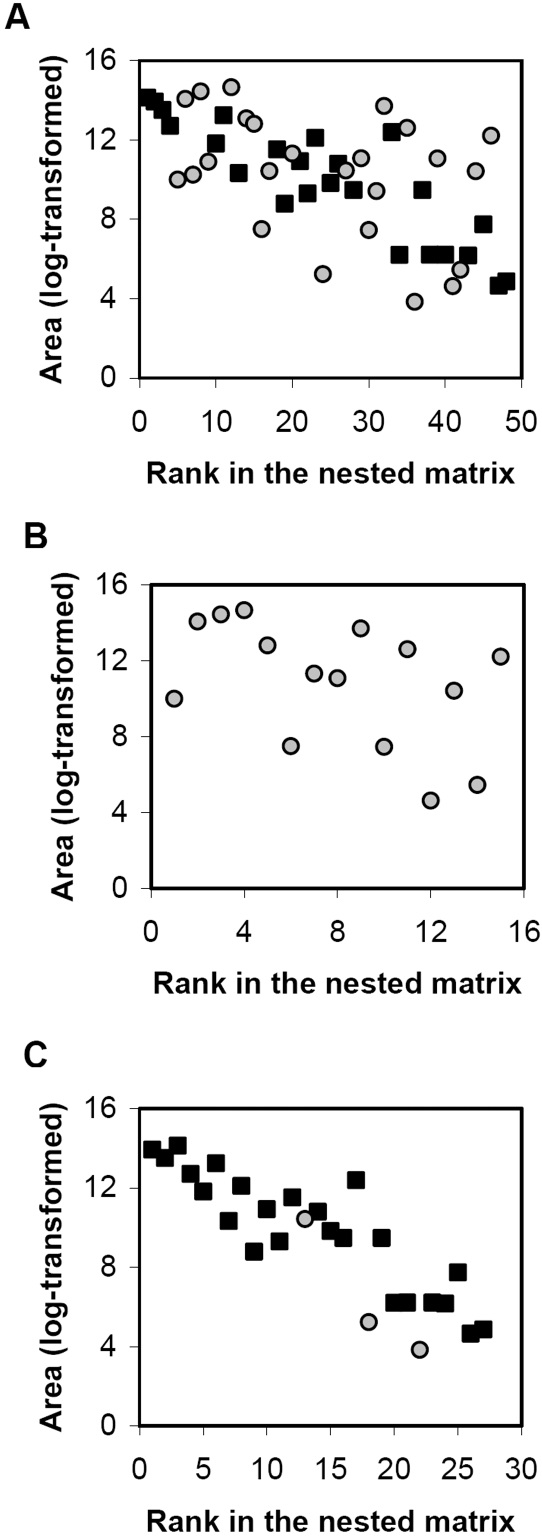
Relationship between the area covered by tree taxa (log-transformed) and their rank in the nested matrices after rearrangement for nestedness. Symbols correspond to tree phyla (gray circles: Magnoliophyta; black squares: Conipherophyta). (A) Largest connected component. (B) Compartment C5. (C) Compartment C6.

As expected, the correlation between the abundance and sampling intensity of tree species was significant and positive (Kendall's rank correlation test; τ = 0.59; p-value<0.001). The correlation between the abundance of given tree taxon and rank in the nested matrix (Fig. 5) was significant and negative for the largest component of the network (Kendall's rank correlation test; τ = −0.45; p-value<0.001) and for compartment C6 (Kendall's rank correlation test; τ = −0.68; p-value<0.001). A similar trend was found for compartment C5 (Kendall's rank correlation test; τ = −0.35; p-value = 0.067). Unsurprisingly, the correlation between the sampling intensity of a given tree taxon and rank in the nested matrix was also significant and negative for the largest component of the network (Kendall's rank correlation test; τ = −0.68; p-value<0.001), for compartment C5 (Kendall's rank correlation test; τ = −0.64; p-value<0.001) and for compartment C6 (Kendall's rank correlation test; τ = −0.76; p-value<0.001).

#### Relationships with the life-history strategy of fungal species

The ranks of the fungal species depended on their life-history strategy, both in the largest network component (Kruskall-Wallis rank sum test; χ^2^ = 7.83, df = 4, p-value = 0.097) and in the C6 compartment (Kruskall-Wallis rank sum test; χ^2^ = 8.63, df = 4, p-value = 0.071). As expected from their large host range, root decay fungi had lower ranks on average (Fig. 4C).

## Discussion

Our analyses revealed that, in French forests, 48 tree taxa (all but 5 identified to species level) and 154 fungal species were fully connected by host/parasite interactions. Consistent with several predictions [5], [41] concerning the topology of antagonistic webs of species, we found that the tree/parasitic fungus network was significantly compartmentalized. Moreover, as expected from previous studies [3], [14], [18], we showed that compartmentalization reflected ancient events in species phylogeny. Our analyses showed that this compartmentalization of host/parasite interactions reflected major phylogenetic splits, but only in host phylogeny. All the tree species of the Conipherophyta phylum (gymnosperms) were grouped within a single compartment, whereas the distribution of the remaining species into the other five compartments paralleled the phylogenetic divisions within the Magnoliophyta group (angiosperms). Thus, on a broad evolutionary scale, two tree species with a similar history are likely to share the same set of parasitic fungal species. This finding parallels that of a recent study on plant/pathogen interactions, which showed that the likelihood of a pathogen infecting two plant species decreased continuously with phylogenetic distance between the plants, even to ancient evolutionary distances [42]. Our results therefore confirm that “the deep evolutionary history of seed plants is mapped by the present day assemblages of exploiters” [36].

A remarkable result of our study is that major splits in parasite phylogeny (in particular, the split between Ascomycota and Basidiomycota) are hardly reflected in the compartmentalization of the network. Thus, our findings suggest that the deep evolutionary history of the fungal species of Dikarya is not mapped by their present parasitic interactions with tree species. This may be due to the very early divergence of the Ascomycota and Basidiomycota phyla, which are assumed to have separated 400 million years ago [43], or even earlier [44]. Molecular analyses and fossil records of plant/fungus associations [45] indicate that plants have had to contend with Ascomycota and Basidiomycota ever since their invasion of the land, perhaps 460 million years ago [43], [46]. Land plants have probably been the main nutrient source for fungi through much of their evolutionary history [43], [47], and many different types of nutrition (mutualist, parasite, saprobe) have evolved [19]. Both Ascomycota and Basidiomycota had probably developed parasitic associations with land plants long before the divergence between Magnoliophyta and Conipherotyta, which occurred 140 to 180 million years ago [48], [49]. It has even been suggested that the common ancestor of the Basidiomycota was a plant parasite [19]. The observed compartmentalization of the tree/parasitic fungus network may therefore be the result of parasitic fungal species splitting into two groups when the Conipherophyta and Magnoliophyta diverged (both groups containing Ascomycota and Basidiomycota species) and the subsequent coevolution of each set of fungal species with its plant phylum.

In addition, our results showed that compartmentalization reflected the current distributional range of tree species [36]. Tree species having overlapping distributional ranges had a tendency to belong to the same compartment. In particular, our analyses suggested that the segregation between Conipherophyta and Magnoliophyta in terms of distributional range may have reinforced the pattern of compartmentalization. Other than in western pine plantations, gymnosperm species are dominant only in alpine regions and in some parts of lower mountain ranges. They therefore tend to be associated with climates in which precipitation levels are high and the annual temperature range is very broad. This grouping together of gymnosperm species may have facilitated host jumps between these species [50], and their occurrence in harsh climates may have prevented interactions with cold-sensitive fungal species. This may account for gymnosperm species having their own set of parasitic fungal species, different from that of angiosperm species. Moreover, the wider geographic range of angiosperm species may account for their associations with parasitic fungi being more diverse than those of gymnosperm species (unlike gymnosperm species, angiosperm species fell into different compartments). Analyses of parasitic fungus assemblages in German forests also revealed a similar trend (see Fig. 1 and Table 1 in the article by Brandle and Brandl [36]). Finally the particular spatial distribution of the Rosaceae tree species—species covering very small total areas but spread across the entire country as isolated trees or small populations—may account for their scattering throughout the network, with three of these species even falling into the compartment containing all the gymnosperm species.

In addition to significant compartmentalization, we also found significant nestedness, with the largest compartments of the network also being significantly nested. Unlike compartmentalization, nestedness did not reflect any consistent phylogenetic signal. Instead, it seemed to reflect the life-history strategies of fungal species and the current abundance of tree species. We found that root decay fungi formed the core of the nested structures, which suggests that the life-history strategy of these fungal species allowed them to expand their host range. The high saprophytic abilities of these species may account for this result: the ability to survive well without a host could have increased their opportunities for and likelihood of host shifts [51]. Moreover, consistent with previous analyses of host-parasite interaction networks [52], we found a trend toward the host species in the core of the nested structures being the most abundant species. Hence, specialist fungal species interact preferentially with abundant tree species. Three different explanations have been proposed by Vazquez and Aizen [9] to account for such an asymmetric pattern of specialization. All of them may apply to the studied tree/fungus network. The first explanation is the selection for specialization on abundant species (because abundant species constitute a more reliable source of reward than rare species with fluctuating populations). Although the relative abundances of tree species changed considerably during the Quaternary Period due to climatic variations [53], the dominant tree taxa (*Quercus robur, Quercus petraea, Fagus silvatica, Pinus sylvestris*) have remained the same during the last thousand years. It may have been easier for specialist fungal parasites to maintain on these abundant hosts than on rarer hosts. Variation in sampling among tree species is a second explanation for the observed pattern. Indeed, we found that the tree species in the core of the nested structures were the most abundant and consequently the most frequently encountered and sampled. This is because DSF foresters report tree/fungus interactions observed during their daily work in the forest, and encounter abundant tree species more often than rare tree species. Rare fungal species had therefore a higher probability to be observed on abundant tree species than on rare tree species. This could account for fungal species having seemingly few interactions preferentially interacting with the tree species currently most abundant in France. The third explanation is that the same kind of sampling bias occurs in nature: fungal “ parasites ‘sample’ abundant hosts more often than rare ones” [52], similarly to DSF foresters. Consequently, abundant tree species interact with a higher number of parasite species than rare tree species. This third explanation is based on the assumption of ecological neutrality at the individual level (i.e. interactions between individuals occur at random). Models based on this assumption generated patterns of specialization which were closed to the observed patterns for several bipartite networks [9], [52].

Overall, our analyses emphasized how the current complexity of ecological networks results from the diversification of the species and their interactions over evolutionary times. They confirmed that the current architecture of ecological networks is not only dependant on recent ecological processes [54], [55]. Compartmentalization analyses suggested that the current architecture of the tree/parasitic fungus network results mainly from ancient speciation events in seed plants. Ancient speciation events in fungi were hardly reflected in the network architecture. Such asymmetries in the phylogenetic signal have recently been found in several plant-animal mutualistic networks [55] and in one host-parasitoid network [54]. Here we proposed that the very early divergence of the major fungal phyla may account for this asymmetrical influence of past evolutionary history. Nestedness analyses suggested that the network architecture has also been shaped by the evolution of host range in fungal species. The influence of these evolutionary processes (i.e. speciation, host range evolution) on the network architecture will be compared to the influence of human-induced changes in a next study. In particular, we will investigate the extent to which the species introduced by human activities during the last centuries (c.a. 30 species equally distributed between trees and fungi) have altered the network architecture.

## Supporting Information

Table S1 List of parasitic fungal species
(0.35 MB PDF)

Table S2 List of forest tree taxa
(0.13 MB PDF)
