# Correction: Architecture of an Antagonistic Tree/Fungus Network: The Asymmetric Influence of Past Evolutionary History

**DOI:** 10.1371/annotation/c03a4beb-a3fa-4b78-8724-b3c8ce28c415

**Published:** 2008-03-19

**Authors:** Corinne Vacher, Dominique Piou, Marie-Laure Desprez-Loustau

Affiliation 1 was published incorrectly. It should appear:

INRA, UMR1202 Biodiversité Gènes et Communautés, Villenave d'Ornon, France

