# Supplementary material for: Architecture of an Antagonistic Tree/Fungus Network: The Asymmetric Influence of Past Evolutionary History
Source: PLoS One. 2008 Mar 5;3(3):e1740. doi: 10.1371/journal.pone.0001740 (PMC2254192; doi:10.1371/journal.pone.0001740)
Supplement: Table S1 — List of parasitic fungal species (0.35 MB PDF) [file pone.0001740.s001.pdf]

Table S1 List of parasitic fungal species

CODE	*Latin name*	PHYLUM	SUBPHYLUM
AMPHLEI	*Amphiporthe leiphaemia*	ASCOMYCOTA	PEZIZOMYCOTINA
APIOERR	*Apiognomonia errabunda*	ASCOMYCOTA	PEZIZOMYCOTINA
APIOVEN	*Apiognomonia veneta*	ASCOMYCOTA	PEZIZOMYCOTINA
ARMICEP	*Armillaria cepistipes*	BASIDIOMYCOTA	AGARICOMYCOTINA
ARMIGAL	*Armillaria gallica*	BASIDIOMYCOTA	AGARICOMYCOTINA
ARMIMEL	*Armillaria mellea*	BASIDIOMYCOTA	AGARICOMYCOTINA
ARMIOST	*Armillaria ostoyae*	BASIDIOMYCOTA	AGARICOMYCOTINA
ASTETIL	*Asteroma tiliae*	ASCOMYCOTA	PEZIZOMYCOTINA
BISCMED	*Biscogniauxia mediterranea var. mediterranea*	ASCOMYCOTA	PEZIZOMYCOTINA
BLUMJAA	*Blumeriella jaapii*	ASCOMYCOTA	PEZIZOMYCOTINA
BOTRCIN	*Botrytis cinerea*[1]	ASCOMYCOTA	PEZIZOMYCOTINA
BOTRDOT	*Botryosphaeria dothidea*	ASCOMYCOTA	PEZIZOMYCOTINA
BOTRSTE	*Botryosphaeria stevensii*	ASCOMYCOTA	PEZIZOMYCOTINA
CALIPIN	*Caliciopsis pinea*	ASCOMYCOTA	PEZIZOMYCOTINA
CENAFER	*Cenangium ferruginosum*	ASCOMYCOTA	PEZIZOMYCOTINA
CERAFIM	*Ceratocystis fimbriata*	ASCOMYCOTA	PEZIZOMYCOTINA
CERAIPS	*Ceratocystis ips*	ASCOMYCOTA	PEZIZOMYCOTINA
CERAMIN	*Ceratocystis minor*	ASCOMYCOTA	PEZIZOMYCOTINA
CHRYABI	*Chrysomyxa abietis*	BASIDIOMYCOTA	PUCCINIOMYCOTINA
CHRYLED	*Chrysomyxa ledi var. rhododendri*	BASIDIOMYCOTA	PUCCINIOMYCOTINA
COLETUS	*Coleosporium tussilaginis f.sp. senecionis-silvatici*	BASIDIOMYCOTA	PUCCINIOMYCOTINA
COLPQUE	*Colpoma quercinum*	ASCOMYCOTA	PEZIZOMYCOTINA
CORIVER	*Coriolus versicolor*	BASIDIOMYCOTA	AGARICOMYCOTINA
CRISDEP	*Cristulariella depraedans*	ASCOMYCOTA	PEZIZOMYCOTINA
CRONFLA	*Cronartium flaccidum*	BASIDIOMYCOTA	PUCCINIOMYCOTINA
CRONRIB	*Cronartium ribicola*	BASIDIOMYCOTA	PUCCINIOMYCOTINA
CRUMSOR	*Crumenulopsis sororia*	ASCOMYCOTA	PEZIZOMYCOTINA
CRYPCAS	*Cryptodiaporthe castanea*	ASCOMYCOTA	PEZIZOMYCOTINA
CRYPCOR	*Cryptostroma corticale*	ASCOMYCOTA	NA
CRYPHYA	*Cryptodiaporthe hystrix*	ASCOMYCOTA	PEZIZOMYCOTINA
CRYPPAR	*Cryphonectria parasitica*	ASCOMYCOTA	PEZIZOMYCOTINA
CRYPPOP	*Cryptodiaporthe populea*	ASCOMYCOTA	PEZIZOMYCOTINA
CYCLMIN	*Cyclaneusma minus*	ASCOMYCOTA	PEZIZOMYCOTINA
CYCLNIV	*Cyclaneusma niveum*	ASCOMYCOTA	PEZIZOMYCOTINA
CYLIDID	*Cylindrocarpon didymum*	ASCOMYCOTA	PEZIZOMYCOTINA
CYTOAMB	*Cytospora ambiens*	ASCOMYCOTA	PEZIZOMYCOTINA
CYTOKUN	*Cytospora kunzei*	ASCOMYCOTA	PEZIZOMYCOTINA
CYTOLEU	*Cytospora leucostoma*	ASCOMYCOTA	PEZIZOMYCOTINA
CYTONIV	*Cytospora nivea*	ASCOMYCOTA	PEZIZOMYCOTINA
DAEDCON	*Daedaleopsis confragosa*	BASIDIOMYCOTA	AGARICOMYCOTINA
DELPABI	*Delphinella abietis*	ASCOMYCOTA	PEZIZOMYCOTINA
DICADRY	*Dicarpella dryina*	ASCOMYCOTA	PEZIZOMYCOTINA
DIPLMUT	*Diplodia mutila*	ASCOMYCOTA	PEZIZOMYCOTINA
DISCCAS	*Discella castanea*	ASCOMYCOTA	NA
DOTHSEP	*Dothistroma septospora*	ASCOMYCOTA	PEZIZOMYCOTINA
ENTOMAM	*Entoleuca mammata*	ASCOMYCOTA	PEZIZOMYCOTINA
EUTYLAT	*Eutypa lata*	ASCOMYCOTA	PEZIZOMYCOTINA
FISTHEP	*Fistulina hepatica*	BASIDIOMYCOTA	AGARICOMYCOTINA
FOMEFOM	*Fomes fomentarium*	BASIDIOMYCOTA	AGARICOMYCOTINA
FOMICYT	*Fomitopsis cytisina*	BASIDIOMYCOTA	AGARICOMYCOTINA
FOMIPIN	*Fomitopsis pinicola*	BASIDIOMYCOTA	AGARICOMYCOTINA

FUSAOXY	*Fusarium oxysporum*	ASCOMYCOTA	PEZIZOMYCOTINA
FUSASOL	*Fusarium solani*	ASCOMYCOTA	PEZIZOMYCOTINA
FUSIQUE	*Fusicoccum quercus*	ASCOMYCOTA	PEZIZOMYCOTINA
GANOADS	*Ganoderma adspersum*	BASIDIOMYCOTA	AGARICOMYCOTINA
GANOAPP	*Ganoderma applanatum*	BASIDIOMYCOTA	AGARICOMYCOTINA
GANORES	*Ganoderma resinaceum*	BASIDIOMYCOTA	AGARICOMYCOTINA
GNOMLEP	*Gnomonia leptostyla*	ASCOMYCOTA	PEZIZOMYCOTINA
GREMABI	*Gremmeniella abietina*	ASCOMYCOTA	PEZIZOMYCOTINA
GYMNFUS	*Gymnopus fusipes*	BASIDIOMYCOTA	AGARICOMYCOTINA
HERPJUN	*Herpotrichia juniperi*	ASCOMYCOTA	PEZIZOMYCOTINA
HETEANN	*Heterobasidion annosum*	BASIDIOMYCOTA	AGARICOMYCOTINA
HYPOFRA	*Hypoxylon fragiforme*	ASCOMYCOTA	PEZIZOMYCOTINA
INONDRY	*Inonotus dryadeus*	BASIDIOMYCOTA	AGARICOMYCOTINA
LACHWIL	*Lachnellula willkommii*	ASCOMYCOTA	PEZIZOMYCOTINA
LAETSUL	*Laetiporus sulphureus*	BASIDIOMYCOTA	AGARICOMYCOTINA
LEPTADU	*Leptoporus adustus*	BASIDIOMYCOTA	AGARICOMYCOTINA
LEPTLUN	*Leptographium lundbergii*	ASCOMYCOTA	PEZIZOMYCOTINA
LEPTPIN	*Leptostroma pinorum*	ASCOMYCOTA	PEZIZOMYCOTINA
LEPTPRO	*Leptographium procerum*	ASCOMYCOTA	PEZIZOMYCOTINA
LIRUMAC	*Lirula macrospora*	ASCOMYCOTA	PEZIZOMYCOTINA
LIRUNER	*Lirula nervisequia var. nervisequia*	ASCOMYCOTA	PEZIZOMYCOTINA
LOPHCON	*Lophodermium conigenum*	ASCOMYCOTA	PEZIZOMYCOTINA
LOPHPIC	*Lophodermium piceae*	ASCOMYCOTA	PEZIZOMYCOTINA
LOPHPIN	*Lophodermium pinastri*	ASCOMYCOTA	PEZIZOMYCOTINA
LOPHSED	*Lophodermium seditiosum*	ASCOMYCOTA	PEZIZOMYCOTINA
MARSBRU	*Marssonina brunnea*	ASCOMYCOTA	PEZIZOMYCOTINA
MELAALL	*Melampsora allii-populina*	BASIDIOMYCOTA	PUCCINIOMYCOTINA
MELACAR	*Melampsorella caryophyllacearum*	BASIDIOMYCOTA	PUCCINIOMYCOTINA
MELALAR	*Melampsora laricis-populina*	BASIDIOMYCOTA	PUCCINIOMYCOTINA
MELAMOD	*Melanconis modonia*	ASCOMYCOTA	PEZIZOMYCOTINA
MELAPIN	*Melampsora pinitorqua*	BASIDIOMYCOTA	PUCCINIOMYCOTINA
MERIGIG	*Meripilus giganteus*	BASIDIOMYCOTA	AGARICOMYCOTINA
MICRALP	*Microsphaera alphitoides*	ASCOMYCOTA	PEZIZOMYCOTINA
MICRJUG	*Microstroma juglandis*	BASIDIOMYCOTA	USTILAGINOMYCOTINA
MONOMON	*Monochaetia monochaeta*	ASCOMYCOTA	PEZIZOMYCOTINA
MYCOCAS	*Mycosphaerella castaneicola*	ASCOMYCOTA	PEZIZOMYCOTINA
MYCODEA	*Mycosphaerella dearnessii*	ASCOMYCOTA	PEZIZOMYCOTINA
MYCOMIC	*Mycosphaerella microsora*	ASCOMYCOTA	PEZIZOMYCOTINA
NECTCIN	*Nectria cinnabarina*	ASCOMYCOTA	PEZIZOMYCOTINA
NECTCOC	*Nectria coccinea*[2]	ASCOMYCOTA	PEZIZOMYCOTINA
NECTDIT	*Nectria ditissima*	ASCOMYCOTA	PEZIZOMYCOTINA
NECTFUC	*Nectria fuckeliana*	ASCOMYCOTA	PEZIZOMYCOTINA
NECTGAL	*Nectria galligena*	ASCOMYCOTA	PEZIZOMYCOTINA
NECTVEU	*Nectria veuillotiana*	ASCOMYCOTA	PEZIZOMYCOTINA
OPHIPIC	*Ophiostoma piceae*	ASCOMYCOTA	PEZIZOMYCOTINA
OPHISER	*Ophiostoma serpens*	ASCOMYCOTA	PEZIZOMYCOTINA
OPHIULM	*Ophiostoma ulmi*[3]	ASCOMYCOTA	PEZIZOMYCOTINA
OUDEMUC	*Oudemansiella mucida*	BASIDIOMYCOTA	AGARICOMYCOTINA
OXYPPOP	*Oxyporus populinus*	BASIDIOMYCOTA	AGARICOMYCOTINA
PESTFUN	*Pestalotiopsis funerea*	ASCOMYCOTA	PEZIZOMYCOTINA
PEZICIN	*Pezicula cinnamomea*	ASCOMYCOTA	PEZIZOMYCOTINA
PHACCON	*Phacidium coniferarum*	ASCOMYCOTA	PEZIZOMYCOTINA
PHAEGAE	*Phaeocryptopus gaeumannii*	ASCOMYCOTA	PEZIZOMYCOTINA
PHAESCH	*Phaeolus schweinitzii*	BASIDIOMYCOTA	AGARICOMYCOTINA

PHELCHR	*Phellinus chrysoloma*	BASIDIOMYCOTA	AGARICOMYCOTINA
PHELHAR	*Phellinus hartigii*	BASIDIOMYCOTA	AGARICOMYCOTINA
PHELIGN	*Phellinus igniarius*	BASIDIOMYCOTA	AGARICOMYCOTINA
PHELPIN	*Phellinus pini*	BASIDIOMYCOTA	AGARICOMYCOTINA
PHELROB	*Phellinus robustus*	BASIDIOMYCOTA	AGARICOMYCOTINA
PHLOACE	*Phloeospora aceris*	ASCOMYCOTA	PEZIZOMYCOTINA
PHOMJUN	*Phomopsis juniperivora*	ASCOMYCOTA	PEZIZOMYCOTINA
PHOMPSE	*Phomopsis pseudotsugae*	ASCOMYCOTA	PEZIZOMYCOTINA
PHOMQUE	*Phomopsis quercella*	ASCOMYCOTA	PEZIZOMYCOTINA
PHYLACE	*Phyllosticta aceris*	ASCOMYCOTA	PEZIZOMYCOTINA
PHYLGUT	*Phyllactinia guttata*	ASCOMYCOTA	PEZIZOMYCOTINA
PLEUOST	*Pleurotus ostreatus*	BASIDIOMYCOTA	AGARICOMYCOTINA
PLEUPSE	*Pleuroceras pseudoplatani*	ASCOMYCOTA	PEZIZOMYCOTINA
PODOAUC	*Podosphaera aucupariae*	ASCOMYCOTA	PEZIZOMYCOTINA
POLLRAD	*Pollaccia radiosa*	ASCOMYCOTA	PEZIZOMYCOTINA
POLYSQU	*Polyporus squamosus*	BASIDIOMYCOTA	AGARICOMYCOTINA
PUCCEPI	*Pucciniastrum epilobii*	BASIDIOMYCOTA	PUCCINIOMYCOTINA
RHABPSE	*Rhabdocline pseudotsugae*	ASCOMYCOTA	PEZIZOMYCOTINA
RHIZKAL	*Rhizosphaera kalkhoffii*	ASCOMYCOTA	PEZIZOMYCOTINA
RHIZMAC	*Rhizosphaera macrospora*	ASCOMYCOTA	PEZIZOMYCOTINA
RHIZOUD	*Rhizosphaera oudemansii*	ASCOMYCOTA	PEZIZOMYCOTINA
RHIZUND	*Rhizina undulata*	ASCOMYCOTA	PEZIZOMYCOTINA
RHYTACE	*Rhytisma acerinum*	ASCOMYCOTA	PEZIZOMYCOTINA
ROSENEC	*Rosellinia necatrix*	ASCOMYCOTA	PEZIZOMYCOTINA
SARERES	*Sarea resinae*	ASCOMYCOTA	PEZIZOMYCOTINA
SAWATUL	*Sawadaea tulasnei*	ASCOMYCOTA	PEZIZOMYCOTINA
SCHICOM	*Schizophyllum commune*	BASIDIOMYCOTA	AGARICOMYCOTINA
SEIRCAR	*Seiridium cardinale*	ASCOMYCOTA	PEZIZOMYCOTINA
SEPTPOD	*Septotis podophyllina*	ASCOMYCOTA	PEZIZOMYCOTINA
SEPTQUE	*Septoria quercicola*	ASCOMYCOTA	PEZIZOMYCOTINA
SIROSTR	*Sirococcus strobilinus*	ASCOMYCOTA	NA
SPARCRI	*Sparassis crispa*	BASIDIOMYCOTA	AGARICOMYCOTINA
SPHASAP	*Sphaeropsis sapinea*	ASCOMYCOTA	PEZIZOMYCOTINA
STEGPYR	*Stegonsporium pyriforme*	ASCOMYCOTA	PEZIZOMYCOTINA
STERHIR	*Stereum hirsutum*	BASIDIOMYCOTA	AGARICOMYCOTINA
STERPUR	*Stereum purpureum*	BASIDIOMYCOTA	AGARICOMYCOTINA
STERSAN	*Stereum sanguinolentum*	BASIDIOMYCOTA	AGARICOMYCOTINA
STIGCAR	*Stigmina carpophila*	ASCOMYCOTA	PEZIZOMYCOTINA
SYDOPOL	*Sydowia polyspora*	ASCOMYCOTA	PEZIZOMYCOTINA
TAPHBET	*Taphrina betulina*	ASCOMYCOTA	TAPHRINOMYCOTINA
TAPHCAE	*Taphrina caerulescens*	ASCOMYCOTA	TAPHRINOMYCOTINA
TAPHCER	*Taphrina cerasi*	ASCOMYCOTA	TAPHRINOMYCOTINA
TAPHPOP	*Taphrina populina*	ASCOMYCOTA	TAPHRINOMYCOTINA
TITATRE	*Titaeosporina tremulae*	ASCOMYCOTA	PEZIZOMYCOTINA
TRAMGIB	*Trametes gibbosa*	BASIDIOMYCOTA	AGARICOMYCOTINA
TRUNHAR	*Truncatella hartigii*	ASCOMYCOTA	PEZIZOMYCOTINA
UREDQUE	*Uredo quercus*	BASIDIOMYCOTA	PUCCINIOMYCOTINA
VALSSOR	*Valsa sordida*	ASCOMYCOTA	PEZIZOMYCOTINA
VENTFRA	*Venturia fraxini*	ASCOMYCOTA	PEZIZOMYCOTINA
VENTINA	*Venturia inaequalis*	ASCOMYCOTA	PEZIZOMYCOTINA
VERTDAH	*Verticillium dahliae*	ASCOMYCOTA	PEZIZOMYCOTINA
ZYTHPIN	*Zythiostroma pinastri*	ASCOMYCOTA	TAPHRINOMYCOTINA

Notes

[1] grouped with *Botryotinia fuckeliana* (anamorphic type [Fermaud M., pers. comm.])

[2] grouped with *Cylindrocarpon candidum* (teleomorphic type [Piou D., pers. comm.])

[3] grouped with *Ophiostoma novo-ulmi* because the two species are only distinguishable by molecular analyses [Piou D., pers. comm.]

NA: non available information
