# Supplementary material for: Architecture of an Antagonistic Tree/Fungus Network: The Asymmetric Influence of Past Evolutionary History
Source: PLoS One. 2008 Mar 5;3(3):e1740. doi: 10.1371/journal.pone.0001740 (PMC2254192; doi:10.1371/journal.pone.0001740)
Supplement: Table S2 — List of forest tree taxa (0.13 MB PDF) [file pone.0001740.s002.pdf]

CODE	*Latin name*	PHYLUM	SUBPHYLUM
ABIEALB	*Abies alba*	CONIFEROPHYTA	PINALES
ABIEGRA	*Abies grandis*	CONIFEROPHYTA	PINALES
ABIENOR	*Abies nordmanniana*	CONIFEROPHYTA	PINALES
ACERGRA	Large Maples *(Acer platanoides, Acer pseudoplatanus)*[1]	MAGNOLIOPHYTA	SAPINDALES
ACERPET	Small Maples *(Acer campestre, Acer monspessulanum, Acer negundo, Acer opalus)*[1]	MAGNOLIOPHYTA	SAPINDALES
ALNUGLU	*Alnus glutinosa*	MAGNOLIOPHYTA	FAGALES
BETUSPP	*Betulus spp (Betula pendula, Betula pubescens)*[1]	MAGNOLIOPHYTA	FAGALES
CARPBET	*Carpinus betulus*	MAGNOLIOPHYTA	FAGALES
CASTSAT	*Castanea sativa*	MAGNOLIOPHYTA	FAGALES
CEDRATL	*Cedrus spp (Cedrus atlantica, Cedrus libani)*[2]	CONIFEROPHYTA	PINALES
CUPRSEM	*Cupressus sempervirens*	CONIFEROPHYTA	PINALES
FAGUSIL	*Fagus silvatica*	MAGNOLIOPHYTA	FAGALES
FRAXSPP	*Fraxinus spp (Fraxinus angustifolia, Fraxinus excelsior)*[1]	MAGNOLIOPHYTA	LAMIALES
JUGLSPP	*Juglans spp (Juglans nigra, Juglans regia)*[1]	MAGNOLIOPHYTA	FAGALES
LARIDEC	*Larix decidua*	CONIFEROPHYTA	PINALES
LARIKAE	*Larix kaempferi*	CONIFEROPHYTA	PINALES
PICEEXC	*Picea excelsa*	CONIFEROPHYTA	PINALES
PICESIT	*Picea sitchensis*	CONIFEROPHYTA	PINALES
PINUBRU	*Pinus brutia (Pinus brutia, Pinus brutia eldarica)*[2]	CONIFEROPHYTA	PINALES
PINUCEM	*Pinus cembra*	CONIFEROPHYTA	PINALES
PINUHAL	*Pinus halepensis*	CONIFEROPHYTA	PINALES
PINULAR	*Pinus nigra laricio*	CONIFEROPHYTA	PINALES
PINUNIG	*Pinus nigra nigra*	CONIFEROPHYTA	PINALES
PINUPINA	*Pinus pinaster*	CONIFEROPHYTA	PINALES
PINUPINE	*Pinus pinea*	CONIFEROPHYTA	PINALES
PINURAD	*Pinus radiata*	CONIFEROPHYTA	PINALES
PINUSTR	*Pinus strobus*	CONIFEROPHYTA	PINALES
PINUSYL	*Pinus sylvestris*	CONIFEROPHYTA	PINALES
PINUTAE	*Pinus taeda*	CONIFEROPHYTA	PINALES
PINUUNC	*Pinus uncinata*	CONIFEROPHYTA	PINALES
PLATHYB	*Platanus hybrida*	MAGNOLIOPHYTA	PROTEALES
POPUCUL	Cultivated Poplars *(Populus trichocarpa, P. canescens, P.alba, P.nigra and their cultivated hybrids)*[2]	MAGNOLIOPHYTA	MALPIGHIALES

POPUTRE	*Populus tremula*	MAGNOLIOPHYTA	MALPIGHIALES
PRUNAVI	*Prunus avium*	MAGNOLIOPHYTA	ROSALES
PSEUMEN	*Pseudotsuga menziesii*	CONIFEROPHYTA	PINALES
QUERILE	*Quercus ilex*	MAGNOLIOPHYTA	FAGALES
QUERPAT	*Quercus petraea*	MAGNOLIOPHYTA	FAGALES
QUERPUB	*Quercus pubescens*	MAGNOLIOPHYTA	FAGALES
QUERPYR	*Quercus pyrenaica*	MAGNOLIOPHYTA	FAGALES
QUERROB	*Quercus robur*	MAGNOLIOPHYTA	FAGALES
QUERRUB	*Quercus rubra*	MAGNOLIOPHYTA	FAGALES
QUERSUB	*Quercus suber*	MAGNOLIOPHYTA	FAGALES
SORBARI	*Sorbus aria*	MAGNOLIOPHYTA	ROSALES
SORBAUC	*Sorbus aucuparia*	MAGNOLIOPHYTA	ROSALES
SORBDOM	*Sorbus domestica*	MAGNOLIOPHYTA	ROSALES
SORBTOR	*Sorbus torminalis*	MAGNOLIOPHYTA	ROSALES
TAXABAC	*Taxus baccata*	CONIFEROPHYTA	PINALES
THUJPLI	*Thuja plicata*	CONIFEROPHYTA	PINALES
TILISPP	*Tilia spp (Tilia platiphyllos, Tilia cordata)* [1]	MAGNOLIOPHYTA	MALVALES
TSUGHET	*Tsuga heterophylla*	CONIFEROPHYTA	PINALES
ULMUSPP	*Ulmus spp (Ulmus minor, Ulmus laevis, Ulmus glabra)* [2]	MAGNOLIOPHYTA	ROSALES

[1] Since areas were not given at the species level in the IFN database, these species were grouped together.
[2] Subspecies or genetic continuum [Piou D., pers.comm.]

Table S2. List of forest tree taxa
